# *Salmonella* – the ultimate insider. *Salmonella* virulence factors that modulate intracellular survival

**DOI:** 10.1111/j.1462-5822.2009.01368.x

**Published:** 2009-09-23

**Authors:** J Antonio Ibarra, Olivia Steele-Mortimer

**Affiliations:** Laboratory of Intracellular Parasites, NIAID, NIH, Rocky Mountain LaboratoriesHamilton, MT, USA

## Abstract

*Salmonella enterica* serovar Typhimurium is a common facultative intracellular pathogen that causes food-borne gastroenteritis in millions of people worldwide. Intracellular survival and replication are important virulence determinants and the bacteria can be found in a variety of phagocytic and non-phagocytic cells *in vivo*. Invasion of host cells and intracellular survival are dependent on two type III secretion systems, T3SS1 and T3SS2, each of which translocates a distinct set of effector proteins. However, other virulence factors including ion transporters, superoxide dismutase, flagella and fimbriae are also involved in accessing and utilizing the intracellular niche.

## Introduction

*Salmonella enterica* are members of the Enterobacteriaceae family of bacteria, a large group of Gram-negative, facultative anaerobes many of which are a normal part of the gut microbiota in the intestines of vertebrates. Although there are well over 2000 serovars of *S. enterica* only a handful are commonly associated with disease in humans, which usually presents as a self-limiting gastroenteritis or the more severe enteric or typhoid fever. *S. enterica* serovar Typhimurium (*S*. Typhimurium) is one of the most frequent causes of food-borne gastroenteritis in humans, and is also an important pathogen of food-producing animals including cattle, pigs and chickens.

*Salmonella* are facultative intracellular bacteria that are found within a variety of phagocytic and non-phagocytic cells *in vivo*. Following intestinal colonization *Salmonella* enter enterocytes, M cells and dendritic cells (DCs) in the intestinal epithelium. Subsequently *Salmonella* that reach the submucosa can be internalized by resident macrophages and rapidly disseminate through the blood stream accumulating in mesenteric lymph nodes and, ultimately, the spleen (Salcedo *et al*., 2001). Altogether the ability of *Salmonella* to survive in a variety of host cells is vital to its success as a pathogen. The large assortment of bacterial and host factors that determine the outcome of infection is summarized here, focusing primarily on serovar Typhimurium.

## Internalization into host cells

**Fig. 1:**
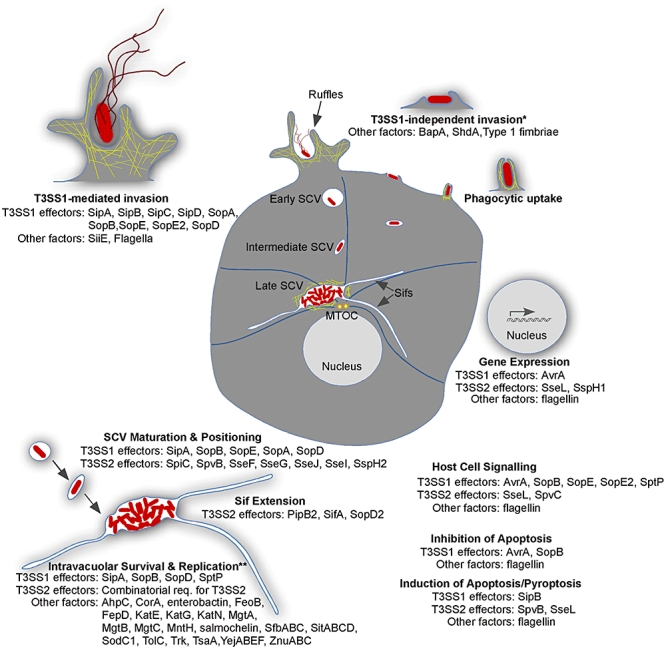
Virulence factors involved in the intracellular survival of *Salmonella*. *Salmonella* can enter host cells by invasion (T3SS1-mediated) or phagocytosis. In addition, a T3SS1-independent invasion* has been shown to occur in several cell types that may be mediated by fimbriae or non-fimbrial adhesins. Following internalization *Salmonella* remain within a modified phagosome known as the SCV (*Salmonella*-containing vacuole). Biogenesis of the SCV and its translocation to the MTOC (microtubule-organizing centre) involves interactions with the host cell endocytic pathway and microtubules and is mediated by a variety of T3SS1 and T3SS2 effector proteins. Survival and replication within the SCV are dependent on a number of factors including nutrient acquisition and avoidance of host antibacterial activities. **Listed are a number of factors implicated but not necessarily proven to be required for intracellular survival. Yellow and blue lines indicate actin, associated with invasion and the SCV, and microtubules, required for positioning of the SCV and Sif extension respectively.

Internalization of *Salmonella* into host cells can occur via at least two distinct processes (Fig. 1). Professional phagocytes such as macrophages utilize phagocytic uptake to efficiently recognize and internalize bacterial pathogens. *Salmonella* can also actively invade both phagocytic and non-phagocytic cells using a type III secretion system (T3SS), T3SS1. Phagocytosis of Gram-negative bacteria is a complex mechanism that involves multiple receptors, some of which increase the efficiency of uptake and others that activate different signalling pathways in the phagocyte. Pattern-recognition receptors recognize pathogen-associated molecular patterns, including lipopolysaccharide (LPS) and flagellin, and binding to ligand, either on the cell surface or inside the phagosome, can affect phagosome maturation, signalling and gene expression (reviewed in Kumar *et al*., 2009). Whereas phagocytosis is an essential innate immune function that has developed to sample a potentially vast array of different pathogens, T3SS1-mediated invasion by *Salmonella* is a highly specific process that depends on the tightly regulated expression of a number of bacterial factors (Takaya *et al*., 2005; Kage *et al*., 2008). In a remarkably co-ordinated process a small group of effector proteins (SipA, SipC, SopB/SigD, SopD, SopE2 and SptP) induce dramatic rearrangement of the actin cytoskeleton resulting in massive localized membrane ruffles and rapid internalization of the bacteria (for review see McGhie *et al*., 2009). In addition to phagocytosis and T3SS1-mediated invasion, fimbriae and/or non-fimbrial adhesins on the surface of *Salmonella* may also mediate attachment and internalization via a T3SS1-independent process (Guo *et al*., 2007).

## The *Salmonella*-containing vacuole

Following internalization *Salmonella* survive and replicate within a modified phagosome known as the *Salmonella*-containing vacuole (SCV), which initially is marked by the accumulation of early endosome markers. These ‘early’ markers are then rapidly removed and within 60–90 min post invasion (p.i.) SCVs become highly enriched in markers of late endosomes and lysosomes particularly lysosomal glycoproteins (Steele-Mortimer *et al*., 1999). Concomitantly, the SCV moves from the cell periphery to a juxtanuclear position at the microtubule-organizing centre (MTOC) (Salcedo and Holden, 2003; Deiwick *et al*., 2006). The onset of intracellular replication is accompanied in some cell types by the appearance of Sifs (*Salmonella*-induced filaments), a network of dynamic membrane tubules that radiate from the SCV (Drecktrah *et al*., 2008).

## *Salmonella* virulence determinants affecting intracellular survival

In addition to two T3SSs, *Salmonella* have a type I secretion system and other factors such as fimbriae, flagella and ion transporters that have important roles in establishing and maintaining the intracellular niche. Many virulence factors are encoded on Salmonella Pathogenicity Islands (SPI) on the chromosome. Most notably, T3SS1 and T3SS2 are encoded on SPI1 and SPI2 respectively. Invasion and early post-invasion processes are modulated by T3SS1, flagella, fimbriae and non-fimbrial adhesins; subsequently the T3SS2 and factors involved in nutrient acquisition and avoidance of antibacterial mechanisms are induced. In reality the system is rather more complex and there is considerable temporal overlap.

### Type I secretion systems

BapA and SiiE are two huge surface-associated proteins that have been implicated in invasion/adhesion and are secreted via dedicated type I secretion systems BapBCD and SiiCDF respectively (Latasa *et al*., 2005; Gerlach *et al*., 2007). SiiE, which has multiple 90-amino-acid repeats and a C-terminal secretion signal, is encoded on SPI4, which is co-regulated with SPI1 (Morgan *et al*., 2007; Main-Hester *et al*., 2008).

### Fimbriae

*Salmonella* have 13 predicted fimbrial loci, many of which are induced *in vivo* and are required for biofilm formation, attachment to host cells and colonization but not intracellular survival *per se* (Humphries *et al*., 2001). The type 1 fimbrial adhesin FimH mediates T3SS1-independent uptake in murine DCs (Guo *et al*., 2007).

### Flagella and flagellin

Flagellar-based motility can increase the invasiveness of *Salmonella* (Schmitt *et al*., 2001), although this remains somewhat controversial especially since flagellin monomers are potent inducers of innate immunity (Franchi *et al*., 2006; Miao *et al*., 2006). In *Salmonella*-infected macrophages flagellin is translocated into the cytosol by T3SS1 resulting in activation of the inflammasome and caspase-1-mediated cell death (pyroptosis) (Ren *et al*., 2006; Miao *et al*., 2007; Sun *et al*., 2007). In the intestinal epithelium flagellin induces inflammation while *inhibiting* apoptosis also via TLR5, but the flagellin must be translocated to the basolateral side of the epithelial cells, since TLR5 is not expressed on the apical surface (Gewirtz *et al*., 2001; Vijay-Kumar *et al*., 2006). Flagella are usually downregulated inside the host, although inside macrophages it has been suggested they may be induced with T3SS1 and used for escape (Sano *et al*., 2007).

### T3SS1

At least 15 effectors can be translocated by T3SS1 into the host cell (reviewed in McGhie *et al*., 2009). Four of these, SopE/SopE2, SopB and SipA, cooperatively induce the actin rearrangements required for invasion but almost all of the others have been implicated in a variety of post-invasion processes, including host cell survival, SCV biogenesis and modulation of the inflammatory response. Accumulating evidence suggests that many effector proteins have multiple activities within host cells. For example, the inositol phosphatase SopB is involved in: invasion, Akt activation, fluid secretion and SCV formation/biogenesis/positioning (Terebiznik *et al*., 2002; Hernandez *et al*., 2004; Drecktrah *et al*., 2005; Knodler *et al*., 2005; Mallo *et al*., 2008; Patel *et al*., 2009). Each of these activities is presumably dependent on the generation of specific phosphoinositides where SopB is localized, namely the plasma and SCV membranes. One intriguing possibility is that intracellular localization of SopB determines its specificity, since the subcellular localization of SopB, and therefore presumably its activity, is controlled by ubiquitination (Knodler *et al*., 2009; Patel *et al*., 2009).

In addition to SopB several other T3SS1 and T3SS2 effector proteins intersect with host cell ubiquitin pathways. The T3SS1 effector AvrA is a member of a family of ubiquitin-like acetyltransferases/cysteine proteases produced by bacterial pathogens including the *Yersinia* effector YopJ. AvrA removes ubiquitin from two inhibitors of the NF-κB pathway, IκBα and beta-catenin, thus inhibiting the inflammatory response (Ye *et al*., 2007; Jones *et al*., 2008), activating beta-catenin signalling (Sun *et al*., 2004) and preventing apoptosis in intestinal epithelial cells (Jones *et al*., 2008). SopA in contrast, another effector that can contribute to invasion (Raffatellu *et al*., 2005), is one of several bacterial effectors that have HECT-like E3 ubiquitin-ligase activity (Zhang *et al*., 2006).

Other T3SS1 effectors implicated in SCV/Sif biogenesis are the tyrosine phosphatase SptP, which dephosphorylates the AAA+ ATPase VCP (Humphreys *et al*., 2009) and is required for switching off ruffle formation following invasion (Fu and Galan, 1999), and SipA which is implicated in SCV morphology and juxtanuclear positioning and has been shown to cooperate with the SPI2 effector SifA (Brawn *et al*., 2007).

### T3SS2

T3SS2 is required for systemic virulence in the mouse and survival within macrophages (Hensel *et al*., 1998). Although the roles of individual T3SS2 effectors remain ill defined, several are involved in SCV positioning and the formation of Sifs that extend from the surface of late SCVs (≥ 6 h p.i.) in epithelial cells. SseF and SseG are required for maintenance of the SCV at the MTOC and intracellular replication (Kuhle and Hensel, 2002; Salcedo and Holden, 2003). SifA is essential for Sif formation, a process apparently linked to SCV membrane integrity since mutants lacking SifA are released into the cytosol (Beuzon *et al*., 2002; Brumell *et al*., 2002). Two other T3SS2 effectors, PipB2 and SseJ, cooperate with SifA via a process involving several mammalian proteins. PipB2 interacts with kinesin light chain, a subunit of the kinesin-1 motor complex that drives anterograde transport along microtubules, recruiting it to the surface of the SCV (Henry *et al*., 2006). This interaction drives the extension of Sif tubules from the juxtanuclear SCV towards the periphery of the host cell (Knodler and Steele-Mortimer, 2005). Recent studies focusing on the interaction between SifA and the mammalian protein SKIP have identified small GTPases as potential targets. Thus SKIP interacts directly with rab9, a GTPase involved in lysosome and late endosome function and positioning, and SifA may displace rab9 from this complex (Barbero *et al*., 2002; Ganley *et al*., 2004; Jackson *et al*., 2008). SifA can also bind directly to rab7 and acts as an exchange factor (GEF) for RhoA, a small GTPase that when activated can increase membrane tubulation and, in the presence of SKIP and the T3SS2 effector SseJ, promotes host membrane tubulation (Harrison *et al*., 2004; Lossi *et al*., 2008; Ohlson *et al*., 2008). In epithelial cells infected with mutants lacking SseJ cholesterol accumulation is increased compared with cells infected with wild-type bacteria, and this is associated with a decrease in intracellular replication (Ruiz-Albert *et al.*, 2002; Nawabi *et al*., 2008). Intriguingly, in cells with abnormally high levels of cholesterol, rab9 is sequestered causing defects in membrane trafficking (Ganley and Pfeffer, 2006), suggesting a possible link exists between SseJ and rab9.

Three T3SS2 effectors interfere with host cell ubiquitin pathways (Quezada *et al*., 2009). SspH 1 and SspH 2 are members of a family of ubiquitin E3 ligases found in pathogenic bacteria including *Shigella* and *Yersinia* (Miao *et al*., 1999; Quezada *et al*., 2009). The function of these two close homologues remains unknown, although SspH 2 colocalizes with actin around the SCV (Miao *et al*., 2003) and it also has a targeting signal for localization at tight junctions in polarized epithelial cells (Quezada *et al*., 2009). SseL is a deubiquitinase that, like AvrA, can modulate NF-κB activation downstream of IκBα kinases although whether it causes suppression or activation of the pathway remains unclear (Coombes *et al*., 2007; Rytkonen *et al*., 2007; Le Negrate *et al*., 2008).

### Virulence plasmid

Two genes, *spvB* and *spvC*, encode the principal factors for plasmid-mediated virulence of serovar Typhimurium (Matsui *et al*., 2001). Both are translocated via the T3SS2 into host cells (Browne *et al*., 2008; Mazurkiewicz *et al*., 2008). SpvB ADP-ribosylates actin, destabilizes the cytoskeleton and is associated with host cell cytotoxicity (Lesnick *et al*., 2001; Tezcan-Merdol *et al*., 2001; Kurita *et al*., 2003; Browne *et al*., 2008). SpvC has phosphothreonine lyase activity and can inactivate the mitogen-activated protein kinases Erk1/2, JNK and p38 in mammalian cells (Li *et al*., 2007; Mazurkiewicz *et al*., 2008).

### Superoxide dismutase

Many host cells produce reactive oxygen species, largely through the activity of the phagosome NADPH oxidase (NOX2) that are required for killing of intracellular pathogens. To counteract this activity *Salmonella* uses a superoxide dismutase, SodCI, to confer protection from extracellular reactive oxygen species. SodCI is ‘tethered’ within the periplasm and protease resistance may be a critical property that allows this enzyme to function in the harsh environment of the phagosome (Krishnakumar *et al*., 2007; Pacello *et al*., 2008).

### Ion acquisition

In the eukaryotic host, iron availability is limited due to the activity of iron-binding proteins such as transferrin and Nramp1 (natural resistance-associated macrophage protein one or Slc11A1), a divalent metal-proton symporter found in macrophages, neutrophils and DCs (Nairz *et al*., 2009). To overcome this limitation *Salmonella* produce two siderophores, enterobactin and salmochelin, in response to iron deprivation (for review, see Muller *et al*., 2009). Salmochelin is a glucosylated derivative of enterobactin and this modification may be important for resistance to lipocalin-2, an antimicrobial protein that prevents bacterial iron acquisition in the inflamed intestinal epithelium (Raffatellu *et al*., 2009). A recent study found that SCVs in macrophages contain enough iron to affect activity of metal-responsive promoters independently of Nramp1 (Taylor *et al*., 2009); however, it is possible that the requirement for iron transporters could change under different conditions, for example when cells are treated with IFN-γ (Nairz *et al*., 2007; Nairz *et al*., 2008). Comparison of mutant Typhimurium strains lacking the iron transporters encoded by *feoB* or *sitABCD* revealed that both are required for survival in Nramp1(−/−) mice and replication in macrophages and that the Nramp1 homologue MntH, which prefers Mn(II) over Fe(II), is also required for optimal virulence (Janakiraman and Slauch, 2000; Boyer *et al*., 2002; Zaharik *et al*., 2004).

*Salmonella* has three distinct systems for uptake of Mg^2+^: CorA, MgtA and MgtB, each of which is essential for virulence (Blanc-Potard and Groisman, 1997; Papp-Wallace *et al*., 2008). In addition, MgtC, encoded on the same operon as MgtB, while not a Mg^2+^ transporter is required for intramacrophage survival and growth in magnesium-depleted medium (Gunzel *et al*., 2006; Alix and Blanc-Potard, 2008).

Two other metal ions implicated in intracellular survival are K^+^ and Zn^2+^. The ZnuABC high-affinity Zn^2+^ uptake system is required for growth of *S*. Typhimurium in low-zinc conditions and intracellularly in some cultured cells and ZnuABC mutants are defective for virulence in both susceptible and resistant mouse strains (Ammendola *et al*., 2007). The Trk system is a multiunit protein complex that functions as a low-affinity K+ transporter and may function in resistance to antimicrobial peptides (Parra-Lopez *et al*., 1994).

## Conclusions

In the last 20 years remarkable progress has been made in our understanding of how *Salmonella* interact with eukaryotic host cells. Nevertheless, many of the most interesting questions remain unanswered. Development of more sophisticated *in vitro* systems such as co-cultures or 3D cultures that more closely recreate the *in vivo* environment is one promising area.
